# Regulation of Early Adipose Commitment by Zfp521

**DOI:** 10.1371/journal.pbio.1001433

**Published:** 2012-11-27

**Authors:** Sona Kang, Peter Akerblad, Riku Kiviranta, Rana K. Gupta, Shingo Kajimura, Michael J. Griffin, Jie Min, Roland Baron, Evan D. Rosen

**Affiliations:** 1 Division of Endocrinology, Beth Israel Deaconess Medical Center, Boston, Massachusetts, United States of America; 2 Harvard Medical School, Boston, Massachusetts, United States of America; 3 AstraZeneca R&D Mölndal, Mölndal, Sweden; 4 Department of Oral Medicine, Infection and Immunity, Harvard School of Dental Medicine, and Endocrine Unit, Massachusetts General Hospital, Boston, Massachusetts, United States of America; 5 Department of Cancer Biology and Division of Metabolism and Chronic Disease, Dana-Farber Cancer Institute, Boston, Massachusetts, United States of America; University of Cambridge, United Kingdom

## Abstract

While there has been significant progress in determining the transcriptional cascade involved in terminal adipocyte differentiation, less is known about early events leading to lineage commitment and cell fate choice. It has been recently discovered that zinc finger protein 423 (Zfp423) is an early actor in adipose determination. Here, we show that a close paralog of Zfp423, Zfp521, acts as a key regulator of adipose commitment and differentiation in vitro and in vivo. Zfp521 exerts its actions by binding to early B cell factor 1 (Ebf1), a transcription factor required for the generation of adipocyte progenitors, and inhibiting the expression of Zfp423. Overexpression of Zfp521 in cells greatly inhibits adipogenic potential, whereas RNAi-mediated knock-down or genetic ablation of Zfp521 enhances differentiation. In addition, *Zfp521^−/−^* embryos exhibit increased mass of interscapular brown adipose tissue and subcutaneous white adipocytes, a cell autonomous effect. Finally, Ebf1 participates in a negative feedback loop to repress Zfp521 as differentiation proceeds. Because Zfp521 is known to promote bone development, our results suggest that it acts as a critical switch in the commitment decision between the adipogenic and osteogenic lineages.

## Abstract 2

Zfp521 is a novel antiadipogenic transcription factor that helps to determine the identity of a mesenchymal cell as bone or fat.

## Author Summary

Adipocytes or fat cells derive from the same stem cells that give rise to bone, cartilage, and muscle. Understanding the switch between bone and fat is of particular medical importance, with implications for diseases like osteoporosis and obesity. Here, we have identified a transcription factor called Zfp521 which is integral to adipogenesis. Prior work has shown that Zfp521 is important in bone formation. Using a variety of overexpression and knockout models, we show that Zfp521 blocks fat cell formation in cultured cells and in living animals. Furthermore, we show that Zfp521 performs this function by binding to another transcription factor, early B cell factor 1 (Ebf1), and repressing the expression of Zfp423, a transcriptional regulator of preadipocyte determination. Finally, we show that Zfp521 is itself repressed by Ebf1, providing a mechanism that turns the levels of this antiadipogenic protein off once the decision to undergo fat cell development has been made. We postulate that Zfp521 regulates one of the earliest events in the lineage switch between bone and fat.

## Introduction

Mesenchymal stem cells can differentiate into a variety of lineages, including fat, bone, muscle, and cartilage [1]. In particular, there has been emphasis placed on understanding how commitment is determined in cells that could become bone or fat, as these processes are considered relevant to the twin epidemics of obesity and osteoporosis. Numerous studies have shown an inverse relationship between bone and fat development, such that marrow adiposity increases with age [2], while the clinical use of proadipogenic PPARγ agonists increases fracture risk [3]–[6]. At the molecular level, reciprocal regulation between adipogenic and osteogenic regulatory factors has been noted. Activation of PPARγ inhibits the activity of the osteoblastogenic master regulator Runx2 [7], while the osteogenic transcription factor Msx2 inhibits PPARγ and C/EBPα [8],[9]. Other transcriptional components that exert opposing effects on adipogenesis and osteogenesis include Taz [10], ΔfosB [11], Rb [12], and Maf [13]. Together, these studies suggest that there is a molecular balance between these two lineages.

Late events in adipocyte terminal differentiation have been well-studied, and a transcriptional cascade has been identified that includes PPARγ, C/EBPα, and a burgeoning network of other factors [14]. Our understanding of early events in adipogenesis, however, is relatively underdeveloped. Notably, Zfp423 and Tcf7L1 have been recently proposed to facilitate early determination events in adipogenesis [15]–[17]. Zfp423 is a large and interesting molecule, containing 30 C2H2 Krüppel-like zinc fingers. In addition to promoting adipose lineage determination, Zfp423 also regulates development in the brain [18],[19], exerting its actions in part through physical interactions with various binding partners, including RAR/RXR [20], Smads [15], and early B cell factor 1 (Ebf1) [21],[22]. Originally described as a regulator of olfactory epithelial differentiation [23] and B-cell lymphopoiesis [24], Ebf1 is proadipogenic [25],[26] and also appears to operate early in the commitment phase of differentiation [27]. Thus, *Ebf1* ^−/−^ mice are not only lipodystrophic but actually lack adipocyte progenitor cells [27],[28].

Zfp521, also known as Evi-3 in the mouse (ZNF521/EHZF in the human), is the only close paralog of Zfp423 in the mammalian genome; both factors contain he same array of 30 C2H2 Krüppel-like zinc fingers. Zfp521 is enriched in primitive cell types, such as neural precursor cells, immature olfactory neurons, and CD34^+^ early hematopoietic progenitors [29]–[31]. Interestingly, Zfp521 is also a critical regulator of osteoblast formation in vivo [32],[33], and in chondrocytes, Zfp521 acts downstream of parathyroid hormone-related peptide to inhibit Runx2 and increase growth plate thickness [32]. The tissue distributions of Ebf1 and Zfp521 are similar [31],[34], and Zfp521 opposes Ebf1 activity in B-cell development [35],[36] and olfactory epithelium development [37]. Taken together, Zfp521 appeared to us to be an excellent candidate for a factor regulating critical early events in adipogenesis.

Here, we identify such a role for Zfp521 as a negative regulator of adipose tissue development. Expression of Zfp521 is reduced during adipogenesis, and gain- and loss-of function studies in vitro and in vivo demonstrate that Zfp521 is a repressor of adipogenesis. We identify Zfp423 as a critical downstream target of Zfp521, and we show that the ability of Zfp521 to repress Zfp423 and adipogenesis depends upon its ability to bind to and inhibit the transcriptional activity of the proadipogenic factor Ebf1. Finally, we establish a negative feedback loop in which Ebf1 binds to an intronic enhancer of the Zfp521 gene and represses its expression. Our data establish that Zfp521 acts early in adipogenesis to negatively regulate differentiation, at least in part through direct inhibition of Ebf1 and subsequent repression of Zfp423 expression.

## Results

### Zfp521 Inhibits Adipogenesis In Vitro

**Figure 1:**
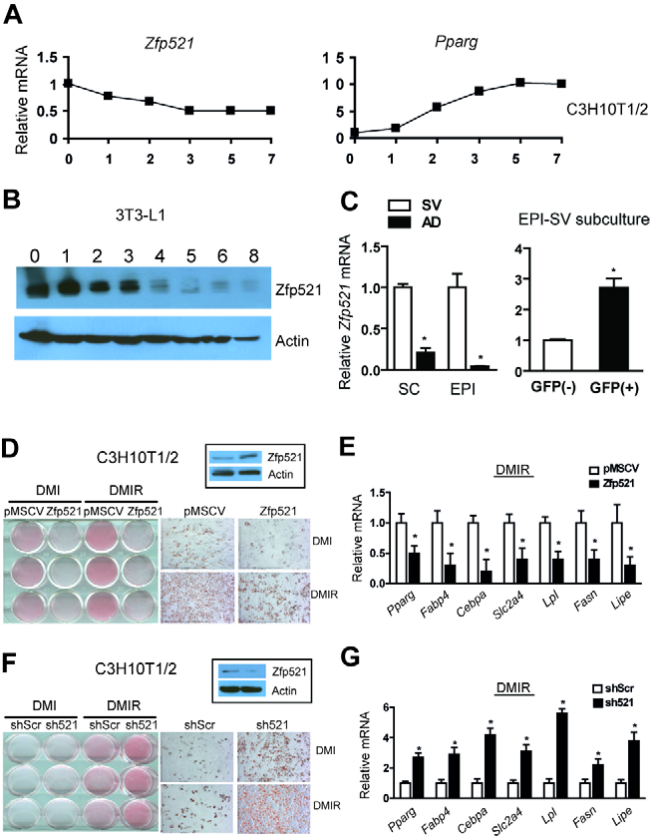
Zfp521 is a suppressor of adipogenesis in vitro. (A) C3H10T1/2 cells were differentiated and RNA isolated at the indicated time points. Gene expression of *Zfp521* and *Pparg* was measured by Q-PCR and normalized to cyclophilin. Data shown as mean of three biological replicates. (B) Protein lysates isolated during 3T3-L1 adipogenesis were subjected to western blotting with anti-Zfp521 antibody. (C, left) Zfp521 mRNA expression was measured in fractionated subcutaneous and epididymal fat tissue taken from wild-type mice (SV, stromal-vascular fraction; AD, adipocytes). (C, right) SV of epididymal fat tissue from Zfp423^GFP^ transgenic mice was sorted with GFP antibody and plated. After washing away floating cells, Zfp521 mRNA expression was measured in GFP− and GFP+ cells. (D–G) C3H10T1/2 cells were retrovirally transduced with Zfp521, empty vector, shRNA specific forZfp521 (sh521), or a scrambled hairpin (Scr). Overexpression and knock-down were confirmed by immunoblotting of Zfp521 prior to differentiation in the boxed insert. Cells were differentiated with DMI or DMI plus rosiglitazone (DMIR) and stained with oil red-O (D, F) and adipocyte markers were determined by Q-PCR (E, G) on day 8. Data presented as mean ± SD, *n* = 3, *p<0.05.

We noted that expression of Zfp521 declines during adipogenesis in multipotent C3H10T1/2 cells (Figure 1A). This pattern was also seen during differentiation of 3T3-L1, 3T3-F442A, and human adipose stromal cells (ASCs) into adipocytes (Figures S1A–S1C), and protein levels declined as well (Figure 1B). Consistent with the in vitro data, Zfp521 levels are higher in the stromal-vascular fraction (which contains preadipocytes) than in the adipocyte fraction of fat pads taken from wild-type mice (Figure 1C). To prove that Zfp521 is expressed in bona fide preadipocytes, we isolated green fluorescent protein (GFP)+ cells from the stromal-vascular fraction of transgenic mice bearing GFP inserted into the Zfp423 start site; such cells have been shown to represent the preadipocyte population of the stromal-vascular fraction (SVF) [16]. As expected, GFP+ cells had significantly higher Zfp521 expression than GFP− cells from the same fat pads (Figure 1C). On the basis of its expression pattern, we predicted that Zfp521 would inhibit adipogenesis. This was indeed the case, as overexpression of Zfp521 in C3H10T1/2 (Figure 1D, 1E), 3T3-L1 cells (Figure S1D, S1E), and mouse embryonic fibroblasts (MEFs) (Figure S2F, S2G) blocked lipid accumulation and adipogenic gene expression, while shRNA-mediated knockdown of Zfp521 (sh521) had the opposite effect (Figures 1F, 1G, S1F, and S1G). This was not an off-target effect of the Zfp521 shRNA, as *Zfp521^−/−^* primary MEFs and stromal-vascular cells taken from the fat pads of *Zfp521^+/−^* mice also showed enhanced adipogenic potential compared to wild-type cells (Figures S1H–S1K). This effect was also true in human ASCs, indicating that the role of Zfp521 as an antiadipogenic factor is not confined to the rodent lineage (Figure S1L, S1M). Furthermore, overexpression of Zfp521 in C3H10T1/2 and MEFs pushed cells towards an osteoblastic fate, demonstrated by staining and gene expression patterns (Figure S2A–S2D). Thus, the combined data from gain- and loss-of-function studies consistently demonstrate that Zfp521 acts as a repressor of adipogenesis and a promoter of osteogenesis in multiple systems in vitro.

### Reducing Zfp521 Enhances Adipogenesis In Vivo

**Figure 2:**
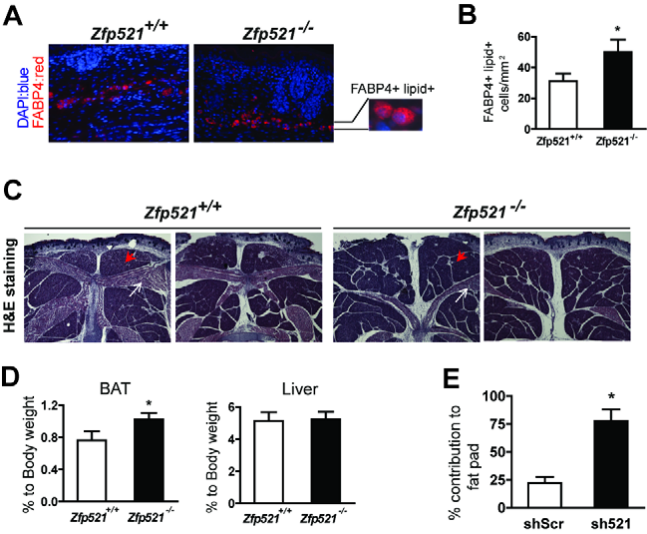
Reduction of Zfp521 enhances adipogenic potential in vivo. (A) Sections of the subcutaneous region of e18.5 *Zfp521^+/+^* and *Zfp521^−/−^* embryos were stained with anti-FABP4 (red) and DAPI (blue). The magnified section shows representative FABP4^+^, lipid-filled cells. (B) ∼20–25 images per embryo were used to quantify the number of lipid-filled and FABP4-positive adipocytes. *Zfp521^+/+^*, *n* = 4 embryos; *Zfp521^−/−^*, *n* = 6 embryos; *p<0.05. (C, D) Representative histology (HE staining) of the interscapular BAT region from *Zfp521^+/+^* and *Zfp521^−/−^* e18.5 embryos (red arrowhead, interscapular BAT; white arrow, skeletal muscle) (C) and BAT and liver weight relative to total body weight (D). Data presented as mean ± SD, *Zfp521^+/+^* (*n* = 9); *Zfp521^−/−^* (*n* = 15), *p<0.05. (E) shZfp521 and scrambled hairpin (shScr) expressing F442A cells were mixed and injected into nude mice. Percentage contribution of shScr and sh521-expressing cells in fat pads determined using specific Q-PCR (see text for details). Data presented as mean ± SD, *n* = 11, *p<0.05.

We next sought to determine whether our findings could be replicated in vivo. Unfortunately, *Zfp521^−/−^* mice die shortly after birth, so we focused our analysis on the development of white and brown adipocytes in late stage embryos (embryonic day 18.5). *Zfp521^−/−^* embryos display an increased number of differentiating white adipocytes in the subdermal connective tissue when detected by the concordance of FABP4 immunostaining and cytoplasmic lipid droplets (Figures 2A, 2B). Remarkably, *Zfp521^−/−^* embryos display significantly enlarged brown adipose depots relative to wild-type littermates when assessed by histology (Figure 2C) and tissue weight (Figure 2D) without major gene expression changes (); liver weight, by contrast, is not different between the two genotypes. Together, these observations suggest that Zfp521 negatively regulates the embryonic development of white and brown adipose tissue.

To determine if these effects were cell autonomous, we took advantage of the ability of 3T3-F442A cells to develop into fat pads when implanted into nude mice [38] to create a competition-based assay for adipogenic potential in vivo. 3T3-F442A cells were transduced with retrovirus expressing either sh521 or a scrambled shRNA control (shScr), and the two cell types were then mixed together at a 1∶1 ratio prior to implantation in nude mice; we reasoned that the hairpin conferring greater adipogenic potential should be overrepresented in the resulting fat pad. Hematoxylin–eosin (HE) staining and protein expression of C/EBPα and FABP4 from implants show that injected cells developed into adipose tissue (Figures S3B, S3C). The relative amount of each hairpin was determined using quantitative PCR (Q-PCR) to measure hairpin-specific sequences normalized to hairpin-independent sequences. By first determining the relative abundance of each hairpin in mixtures of input cells at fixed ratios, we established a baseline by which we could normalize results from the harvested tissue (Figure S3D). This analysis revealed that sh521 cells accounted for approximately 78% of the adipocytes in the fat pads, significantly greater than the 50% expected if Zfp521 knockdown had no effect (Figure 2E). We thus established that the effect of Zfp521 on adipogenesis is cell autonomous.

### Zfp521 Is an Early Regulator of Adipogenesis

We next sought to determine whether Zfp521 was acting early, late, or perhaps at multiple time points in the differentiation process. As shown previously, Zfp521 sharply repressed lipid accumulation and adipocyte-specific gene expression in C3H10T1/2 cells; this effect was largely reversed by adding PPARγ or C/EBPα (Figures S3E, S3F). We also assessed whether knocking down Zfp521 in cells lacking PPARγ would allow adipogenesis to proceed. While viral delivery of shZfp521 promoted adipogenesis in cells that contain PPARγ (*Ppar*γ*^+/−^*), cells lacking PPARγ entirely (*Ppar*γ^−/−^) did not undergo differentiation (Figures S3G–S3K). Taken together, these data suggest that Zfp521 acts prior to PPARγ in the differentiation cascade.

### Zfp521 Inhibits Preadipocyte Commitment Factor Zfp423

**Figure 3:**
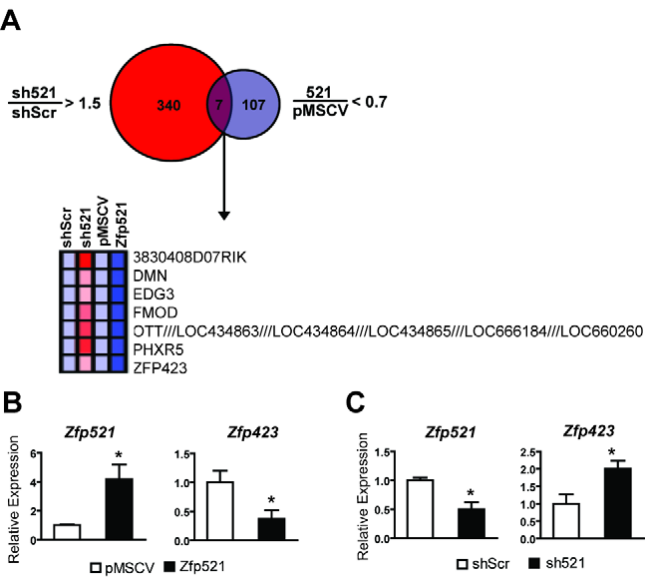
Zfp521 suppresses Zfp423 expression. (A) 3T3-L1 preadipocytes were transduced with retrovirus expressing sh521, shScr, Zfp521, or empty vector. After puromycin selection, RNA was collected and submitted for analysis using Affymetrix arrays. The Venn diagram shows the number of genes up-regulated by sh521 (sh521/shScr>1.5-fold) and down-regulated by Zfp521 (Zfp521/pMSCV<0.7-fold). The heat map corresponds to genes in the intersecting set. (B) Overexpression of Zfp521 in C3H10T1/2 cells represses Zfp423 expression by Q-PCR. (C) Knockdown of Zfp521 in C3H10T1/2 cells enhances Zfp423 expression by Q-PCR. Data presented as mean ± SD, *n* = 3, *p<0.05.

Because Zfp521 is a transcriptional regulator, we sought to identify downstream targets in preadipocytes using Affymetrix arrays. We performed these experiments in both gain-of-function and loss-of-function contexts to enhance our ability to identify bona fide targets of Zfp521, focusing on genes that showed coordinate regulation between Zfp521 overexpression and knockdown. There were 631 genes whose expression fell below 70% of baseline when Zfp521 was targeted by RNAi, while surprisingly few targets (five) increased by more than 1.5-fold when Zfp521 was overexpressed (Figure S4A). A total of three genes (*Dusp1*, *Fos*, and *Serpina3m*) showed coordinate regulation, demonstrating that they are bona fide positive targets of Zfp521. Conversely, there were 347 genes whose expression increased upon treatment with sh521, and 114 that were diminished by Zfp521 overexpression (Figure 3A); only seven of these genes were coordinately regulated. These seven genes include poorly annotated transcripts of uncertain function (e.g. *3830408D07RIK*), some of which reside in a 1.3-MB cluster on the X chromosome (*OTT/LOC434863/LOC434864/LOC434865/LOC666184/LOC660260*). Most of the others are also relatively unstudied, and include a sphingosine-1-phosphate receptor (*Edg3*), a per hexamer repeat gene (*Phxr5*), and the cell structural and matrix genes synemin (*Dmn*) and fibromodulin (*Fmod*). The final gene on the list of those coordinately regulated by Zfp521 was, however, of particular interest to us: Zfp423, the most highly paralogous gene to Zfp521 and a factor recently shown to promote adipose linage commitment [15]. Q-PCR analysis in preadipocytes confirmed that Zfp521 exerts strong repressive effects on Zfp423 expression (Figure 3B), and that reducing Zfp521 has the converse effect (Figure 3C).

### Zfp521 Inhibits Ebf1 Transcriptional Activity through Physical Interaction

**Figure 4:**
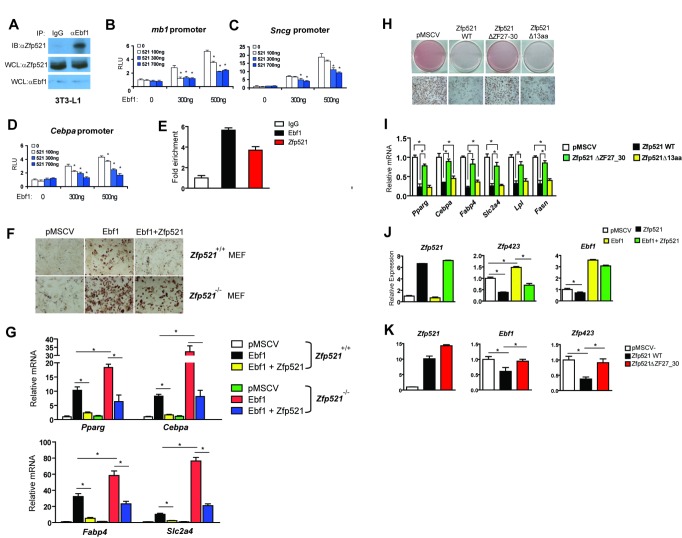
Zfp521 inhibits Ebf1 transcriptional activity through physical interaction. (A) 3T3-L1 preadipocytes were harvested and endogenous Ebf1 was immunoprecipitated using anti-Ebf1 beads and blotted with normal goat-IgG or anti-Zfp521. (B–D) 3T3-L1 preadipocytes were co-transfected with vectors expressing Flag-Zfp521, Myc-Ebf1, and various reporter plasmids containing the *mb1*-promoter (B), *Sncg*-promoter (C), or *Cebpa*-promoter (D). At 24 h after transfection, luciferase activity was normalized to β-galactosidase activity. Data presented as mean ± SD, *n* = 4, *p<0.05. (E) 3T3-L1 preadipocytes were stably transduced with Flag-Ebf1 or Flag-Zfp521. ChIP assay was performed on 3T3-L1 cells that were treated with DMI for 1 h using anti-Flag antibody or an IgG control using PCR primers directed at regions of the Cebpa containing the putative Ebf sites. (F, G) Immortalized *Zfp521^+/+^* and *Zfp521^−/−^* MEFs were transduced with a retrovirus expressing Ebf1, Zfp521, or empty vector and differentiated prior to staining with oil red-O after 8 d (F) and adipocyte gene expression was measured by Q-PCR (G). (H, I) C3H10T1/2 cells were transduced with a retrovirus expressing Zfp521WT, Zfp521ΔZF27-30, Zfp521Δ13aa, or empty pMSCV vector. Cells were differentiated with DMIR and stained with oil red-O and gene expression was measured on day 6. (J) Zfp521 and Ebf1 were expressed in C3H10T1/2 cells alone or in combination, and expression of Zfp521, Ebf1, and Zfp423 was determined by Q-PCR. (K) Zfp521, Zfp521Δ27-30, or pMSCV was expressed in C3H10T1/2 cells and expression of Zfp521, Ebf1, and Zfp423 was determined by Q-PCR. Data presented as mean ± SD, *n* = 3, *p<0.05.

Zfp521 interacts with Ebf1 in the context of hematopoiesis [36]. Because we have previously shown that Ebf1 is a strong inducer of adipogenesis [25],[26], we asked whether Zfp521 interacts with Ebf1 in preadipocytes, and whether this might underlie the anti-adipogenic actions of Zfp521. Zfp521 physically interacts with co-transfected Ebf1 in HeLa cells (Figure S4B), and this effect is also seen using co-immunoprecipitation of native proteins in 3T3-L1 preadipocytes (Figure 4A). Zfp521 inhibits the transcriptional activity of Ebf1 on the mouse mb-1 promoter region as well as the promoters of *Sncg* (a highly Ebf1-responsive gene in adipocytes; MJG and EDR, unpublished data) and *Cebpa* (Figure 4B–4D). Further, Zfp521 is recruited to the same location as Ebf1 on the *Cebpa* promoter [25] (Figure 4E). Ebf1 shows a slight ability to promote differentiation in immortalized *Zfp521^+/+^* MEFs, an effect dramatically enhanced in *Zfp521^−/−^* cells. Reintroduction of Zfp521 reversed the effect in both genotypes (Figure 4F, 4G). Zfp423 interacts with Ebf1 via its most C-terminal cluster of zinc fingers [32]. We therefore tested this in Zfp521 using a mutant allele missing zinc fingers 27–30 (Zfp521ΔZF27-30) (Figure S4D). Zfp521ΔZF27-30 is less able to interact with Ebf1 than wild-type Zfp521 in a co-immunoprecipitation assay (Figure S4E), and exhibits reduced ability to suppress Ebf1 transcriptional activity (Figure S4F). We also focused on the first 13 amino acids of Zfp521 (Figure S4D), which contain a motif conserved among other zinc finger transcriptional repressors like Fog-1 and Fog-2 that is required for Zfp521 to regulate erythropoiesis through physical interaction with GATA-1 and the nuclear remodeling and histone deacteylation (NuRD) complex [32]. In contrast to the ΔZF27-30 mutant, Zfp521Δ13aa is almost fully able to suppress Ebf1 activity on the Cebpa promoter (Figure S4F). Consistent with these results, wild-type Zfp521 and Zfp521Δ13aa, but not Zfp521ΔZF27-30, could fully repress adipogenesis in C3H10T1/2 cells (Figure 4H, 4I). Importantly, Ebf1 induces Zfp423 expression, an effect which can be blocked by WT Zfp521, but not Zfp521ΔZF27-30 (Figures 4J, 4K). This indicates that the effect of Zfp521 on Zfp423 expression is mediated via the interaction with Ebf1.

Given that Zfp521 and Zfp423 contain highly homologous Ebf1 binding domains, we speculated that Zfp521 might compete with Zfp423 for binding to Ebf1. Using coimmunoprecipitation assays, we confirmed that both Zfp521 and Zfp423 bind readily to Ebf1, but to our surprise, the addition of either Zfp521 or Zfp423 enhances binding of the other protein (Figure S5A), an effect seen even in the presence of a large excess of either protein. This suggested to us that Zfp521 and Zfp423 might heterodimerize, and this is indeed the case, even in the absence of Ebf1 (Figures S5B, S5C). The full significance of this interaction is still unclear, but it suggests that Ebf1 binds Zfp521 and Zfp423 simultaneously, with the heterodimer showing enhanced affinity for Ebf1 relative to Zfp521 or Zfp423 monomers or homodimers. We postulate that the downstream effect of Zfp binding to Ebf1 depends upon the content of Zfp521 in the dimer. Consistent with this idea, the Zfp521/Zfp423 ratio falls dramatically almost immediately upon the induction of differentiation, and becomes even more lopsided in favor of Zfp423 as adipogenesis proceeds (Figure S6).

### Zfp521 and Ebf1 Negatively Regulate the Expression Each Other during Adipogenesis

**Figure 5:**
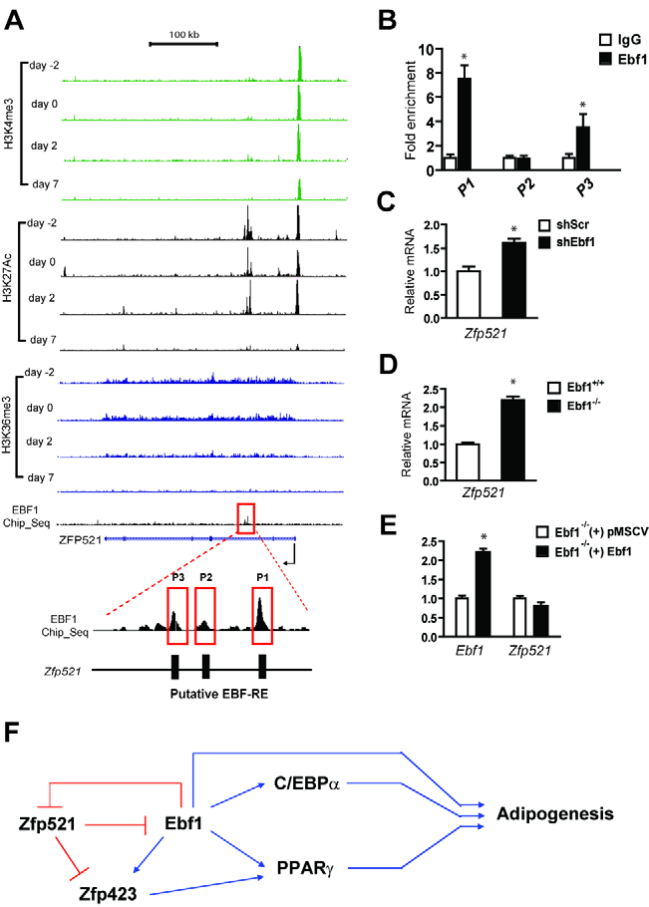
Ebf1 suppresses *Zfp521* expression via an intronic binding site. (A) ChIP-Seq tracks corresponding to the Zfp521 locus are shown for three histone marks: H3K4me3 (green), H3K27Ac (black), and H3K36me3 (blue) at four time-points during 3T3-L1 adipogenesis. A cluster of Ebf1 peaks highlighted in the red box contains three putative Ebf1-responsive elements (EBF-RE). (B) ChIP-PCR analysis was performed with antibody against IgG or Ebf1 in 3T3-L1 preadipocytes. Immunoprecipitated DNA was amplified with Q-PCR using primers designed for the three putative EBF-REs shown in (A). (C) Zfp521 mRNA expression was measured in 3T3-L1 preadipocytes transduced with shScr or shEbf1. (D) Zfp521 mRNA expression was measured in confluent *Ebf1^+/+^* and *Ebf1^−/−^* MEFs. (E) Ebf1 and Zfp521 mRNA expression was measured in *Ebf1^+/+^* and *Ebf1^−/−^* MEFs transduced with Ebf1 or empty vector. Data presented as mean ± SD, *n* = 3, *p<0.05. (F) A proposed model for the transcriptional cascade involving Zfp521, Ebf1, and Zfp423.

In order to determine why Zfp521 levels decline during adipogenesis, we turned to our previously published genome-wide chromatin state maps generated from 3T3-L1 cells [39], which demonstrated a single strong H3K4me3 peak at the Zfp521 promoter that diminishes as differentiation proceeds (Figure 5A). H3K36me3, a mark of transcriptional elongation, also diminishes in a differentiation-dependent manner. Non-promoter H3K27 acetylation (H3K27Ac), which marks active regulatory sites, revealed a single cluster of peaks in the second intron of the Zfp521 gene, which was dramatically reduced in mature adipocytes, suggesting negative regulation at this locus. We have recently profiled Ebf1 binding sites in mature L1 adipocytes and have found a cluster of peaks that precisely coincide with these enhancer sites (Figure 5A). Using ChIP-PCR, we could confirm Ebf1 binding to two of these peaks (Figure 5B). Zfp521 levels rise in 3T3-L1 cells after transduction of shEbf1 (Figure 5C), and are elevated in *Ebf1^−/−^*MEFs (Figure 5D). Furthermore, restoring Ebf1 levels in these cells represses Zfp521 (Figure 5E). These data indicate a role for Ebf1 as a negative regulator of Zfp521 gene expression.

## Discussion

Here we have identified Zfp521 as a potent repressor of adipogenesis in vitro and in vivo. Zfp521 acts early in the cascade of terminal adipocyte differentiation. Our group previously showed that Ebf1 promotes adipogenesis by inducing the expression of PPARγ and C/EBPα [25] and a recent study showed that Ebf1 is required for early commitment of the adipose lineage [28]. Gupta et al. showed that Zfp423 acts as a preadipocyte commitment factor [15]. Zfp521 acts upstream, to inhibit the expression of Zfp423, and also inhibits the expression and function of Ebf1 through physical interaction. As differentiation proceeds, Ebf1 represses the expression of Zfp521 via direct binding to an intronic enhancer (see Figure 5F for a model). Therefore, our data suggest that Zfp521 participates in a feedback loop during adipose commitment and differentiation.

Interestingly, although the anti-adipogenic effects of Zfp521 were demonstrated in every model we tested, these actions were consistently most dramatic in C3H10T1/2 cells. C3H10T1/2 cells are the most primitive in vitro model that we employed, with multi-lineage potential. This observation is consistent with reports that Zfp521 promotes bone and cartilage development in vivo [32]. In chondrocytes, Zfp521 acts downstream of parathyroid hormone-related peptide (PTHrP) to inhibit Runx2 and increasing cellular proliferation and subsequent growth plate thickness [32]. The published data on Zfp521 in bone development have been less clear, with discordance seen between in vitro (where Zfp521 inhibits osteogenesis) and in vivo (where overexpression of Zfp521 increases bone mass) studies [33], and it has been proposed that inhibition of Runx2 by Zfp521 may have differential effects on bone development depending upon timing [33]. Our own data support a role for Zfp521 as a pro-osteogenic factor in vitro (Figures S2A–D). The actions of Zfp521 on osteogenesis and adipogenesis place this factor among a group of transcription factors recently discovered to regulate the relative abundance of these two lineages. These factors, which include Rb, Maf, fosB, Taz, and Ebf1 [10]–[13],[33], have been the subjects of intense inquiry given obvious links to two human diseases of great importance, osteoporosis and obesity.

How does Zfp521 inhibit adipogenesis? Our data point strongly to a mechanism involving the transcriptional repression of the proadipogenic factor Zfp423, although other targets are certainly possible. Relatively few genes were concordantly regulated by knockdown and overexpression of Zfp521 in preadipocytes, and most have no obvious link to adipocyte differentiation. An exception is *Dusp1*, which encodes Map kinase phosphatase-1 (MKP-1). However, MKP-1 has been shown to promote adipogenesis [40], while we found it to be positively regulated by Zfp521. Other target genes are likely to be involved, such as *Cebpa*, which exhibit subthreshold levels of change on the microarray. Zfp521 may have intrinsic DNA-binding activity [22], and some actions may well be mediated by direct effects on the expression of critical developmental genes, although this remains to be proven. We show, however, that binding and inhibition of Ebf1 is a major mechanism by which Zfp521 inhibits adipogenesis. A mutant allele of Zfp521 that cannot bind Ebf1 has greatly reduced ability to repress differentiation. We posited that a simple competition could exist between Zfp423 and Zfp521 for Ebf1, given the high degree of similarity in their C-terminal zinc finger domains. In fact, this proved not to be the case, with Zfp521 and Zfp423 enhancing each others' binding to Ebf1. Furthermore, Zfp521 and Zfp423 can bind directly to one another, suggesting that they form heterodimers that inhibit Ebf1 action. In this vein, we propose that Ebf1 action is contingent upon the relative amounts of Zfp521 and Zfp423 in the resulting complex, although this needs to be formally tested. Finally, this could explain the small amount of residual antiadipogenic activity seen with the Zfp521ΔZF27-30 mutant, which may bind to Zfp423 despite being unable to bind Ebf1. Zfp423 has Smad-binding properties, which have been implicated in its proadipogenic effects [15], and binding directly to Zfp521 could attenuate this activity. We have not investigated whether additional antiadipogenic actions of Zfp521 could be due to effects on the Smad pathway.

Our data thus add to our knowledge of early events in mesenchymal differentiation, and may be an exploitable angle in the battle to promote metabolic and skeletal health.

## Materials and Methods

### Cell Culture

3T3-L1 preadipocytes were obtained from ATCC and maintained in DMEM with 10% calf serum.

HeLa cells, 3T3-F442A cells, and MEFs were maintained in DMEM with 10% FBS. C3H10T1/2 cells were grown and differentiated in αMEM with 10% FBS. Primary *Zfp521* ^+/+^and *Zfp521* ^−/−^ MEFs were generated and immortalized as described previously [41]. For adipose differentiation, cells were induced with a cocktail of dexamethasone (1 µM), insulin (5 µg/ml), and isobutyl methylxanthine (0.5 mM) (DMI) beginning 2 d after confluence was reached (day 0). For MEFs and C3H10T1/2 cells, rosiglitazone (Rosi, 100 nM∼5 µM) was added to the DMI cocktail (DMIR). After 2 d, media was changed to DMEM/FBS plus insulin (5 µg/ml) for an additional 2 d and thereafter maintained until harvest in DMEM/FBS only. Human adipose derived stromal cells (hASCs) were obtained from Jeff Gimble and were maintained in MesenPro media (Invitrogen); cells were differentiated as described previously [42]. Primary MEFs were isolated on embryonic day 13.5 and differentiated with DMI plus Rosi. For osteoblastogenesis, confluent cells were fed with a-MEM media containing β-glycerophophate (2 mM) and ascorbic acid (50 µg/ml) every other day.

### Plasmids

Cebpa (−534/116) and Sncg (−2647/−1468) promoter constructs were generated by ligating PCR amplicons into pGL4 basic (Promega). 6-myc Ebf1 and 3×mb-1 reporter construct were obtained from M. Sigvardsson [43]. 2HA-Zfp521-pCMV, 3Flag-Zfp423-pBICEP, and 3Flag-Zfp521-pBICEP were subcloned at the SalI/BamHI sites. Oligonucleotides representing specific hairpin sequences forZfp521 or a scrambled control were ligated into pSiren-RetroQ (Clontech) at the BamHI/EcoRI sites. To construct WT-Zfp521-pMSCV and Zfp521ΔZF27-30-pMSCV, wild-type or zinc finger 1∼26 regions were PCR amplified with primers containing flanking BamHI and SalI sites and subcloned into pMSCV at the BglII and XhoI sites.

### Retrovirus Preparation and Infection

Retroviral preparation and infection were performed as previously described [44]. Briefly, pMSCV-based overexpression constructs and pSIREN-based knockdown constructs were cotransfected with gag-pol and VSV-G-expressing plasmids into Phoenix packaging cells. 2 d after transfection, viral supernatant was harvested, filtered through 0.45-µm filters, and added to target cells for 12 h along with 8 µg/ml Polybrene. Cells were selected with 4 µ/ml puromycin or 400 µg/ml hygromycin until control cells transduced with a virus carrying no antibiotic resistance cassette were all killed.

### RNA Extraction and Q-PCR

Total RNA was extracted from cells or tissues using TRIzol reagent according to the manufacturer's instructions. cDNA was reverse-transcribed from 2 µg of RNA by using the RETROscript first strand synthesis kit (Ambion). Q-PCR was performed with SYBR Green QPCR Master Mix (Applied Biosystems) using a 7900HT Fast Real-Time PCR System (Applied Biosystems). The relative amount of mRNA normalized to cyclophilin B was calculated by using the delta-delta method [45].

### Co-immunoprecipitation and ChIP-PCR Studies

Co-immunoprecipitation (Co-IP) was performed according to a modified protocol as previously described [44]. Briefly, HeLa and 293T cells were transfected with various DNA constructs using calcium phosphate or Lipofectamine (Invitrogen). A day after transfection, cells were lysed with Triton X lysis buffer (BostonBioProducts) containing 50 mM Tris-HCL, pH 7.4, 150 mM NaCl 1% Triton X-100, 5 mM EDTA, plus protease inhibitors (Complete mini, Roche). 500 µg of protein was incubated with the appropriate antibodies overnight. The next day, protein A/G beads (Santa Cruz Biotechnology) were added and incubated for 1 h, washed with lysis buffer five times and PBS once. Beads were eluted with non-reducing SDS/PAGE loading buffer and subjected to SDS/PAGE and Western blotting. For ChIP-PCR, genomic DNA was extracted, and IP was performed according to the manufacturer's protocol (Upstate Biotechnology). Cross-linking was performed as described in the manufacturer's protocol. DNA was sheared using a sonic dismembrator model 100 (Fisher Scientific) at half maximum speed for 10 s. IP was performed using 2 µg of anti-Flag (Sigma), anti-Ebf1 (Abcam), or IgG (Sigma).

### Western Blot Analysis

Whole-cell protein lysates were prepared according to the manufacturer's protocol using Triton-X lysis buffer and protease inhibitor cocktail. 20–30 µg of protein was resolved using 4%–20% Tris-glycine gradient gels and transferred to PVDF membrane. After blocking with 5% non-fat dried milk in PBS-Tween (0.25%), membranes were incubated with the appropriate primary antibodies against HA (Covance), FLAG (Sigma), FABP4, MYC, C/EBPα (Santa Cruz Biotechnology), or ZFP521. Anti-goat and anti-mouse or rabbit IgG–peroxidase conjugate (Sigma) were used to detect primary antibodies.

### Reporter Assays

Reporter plasmids were co-transfected with 6Myc-Ebf1, 3Flag-Zfp521-pBICEP, WT-Zfp521-pMSCV, or Zfp521ΔZF27-30-pMSCV to 3T3-L1 preadipocytes by using Lipofectamine 2000 or Amaxa nucleofactor (Lonza). Beta-galactosidase expression vector (50 ng) was cotransfected to normalize transfection efficiency. Statistics were performed using the Student's *t* test.

### Implantation of 3T3-F442A Cells

3T3-F442A cells were transduced with scrambled (Scr) or shZfp521 expressing vectors. For transplantation, 10^7^ shScr and shZfp521 expressing cells were mixed together in 200 µl PBS and subcutaneously implanted (2×10^7^cells per site) to the sternum of 10–14-wk-old male nude mice (Crl:NU/NU-nuBR, Taconic). An aliquot of the mixed cell population was saved at −80°C for quantification of input ratio. 10 wk after transplantation, fat pads were carefully dissected, and either fixed in buffered formalin or stored at −80°C for genomic DNA and protein isolation. For quantification of the relative abundance of Scr versus shZfp521 cells from transplants, 50 ng of genomic DNA was used to amplify scrambled-, shZfp521-specific, or common vector sequences (see Table S1).

### Animals

*Zfp521^+/−^* mice were obtained from Soren Warming. Animal studies were performed in accordance with the Institutional Animal Care and Use Committee (IACUC) of the Beth Israel Deaconess Medical Center.

### Assessment of Embryonic Development of Adipose Tissue

*Zfp521* ^+/−^ male and female mice were mated on day 0 and males were removed early day 1. On embryonic day 18.5, pregnant females were euthanized and embryos were dissected. Body weight, liver, and interscapular brown adipose tissue weight of individual embryos was measured. Tails were saved for genotyping and embryos were fixed with 4% paraformaldehyde. The next day embryos were washed with PBS, stored in 70% ethanol, and embedded in paraffin and sectioned for HE staining and immunostaining with FABP4 antibody. For secondary antibody, anti rabbit Alexa 647 (Invitrogen) was used along with the FX enhancer (Invitrogen) and Prolong Gold Anti-Fade Reagent with DAPI (Invitrogen) according to the manufacturers instructions. The number of adipocytes was measured by counting cells that were both lipid-filled and FABP4 positive. To detect Zfp521 mRNA expression in Zfp423-marked preadipocytes, epididymal fat tissue from Zfp423^GFP^ mice was fractionated and sorted as described in [16].

### Statistical Analyses

All results are presented as means standard deviations. Statistical difference was determined using Student's *t* test.

## Supporting Information

Figure S1 **(A, B) 3T3-L1 cells (A), 3T3-F442A cells (B), and human ASCs (C) were differentiated and RNA isolated at the indicated time points.** Gene expression ofZfp521/ZNF521 and Pparg/PPARG was measured by Q-PCR. Relative expression was normalized to cyclophilin. Data shown as mean of three biological replicates. (C, D) Retrovirally transduced 3T3-L1 cells expressing Zfp521, empty vector, shRNA specific forZfp521 (sh521) or a scrambled hairpin (Scr) were differentiated with DM or DMI and stained with oil red-O (D, F) and adipocyte markers were determined by Q-PCR (E, G) on day 8. (H, I) Primary MEFs were generated from *Zfp521^+/+^* and *Zfp521^−/−^*embryos and differentiated with DMIR stained with oil red-O (H) and adipocyte markers were determined by Q-PCR (I) on day 8. (J, K) Stromal vascular cells were isolated from epididymal adipose tissue of *Zfp521^+/+^* and *Zfp521^+/−^* mice. Cells were differentiated with DMIR, stained with oil red-O (J) and adipocyte gene expression was determined by Q-PCR (K) on day 8. Data presented as mean ± SD, *n* = 3, *p<0.05. (L, M) Human ASCs were transfected with Zfp521 or pMSCV and differentiated with DMIR. Adipogenesis was assessed with oil red-O and gene expression analysis on day 7.
(TIF)

Figure S2 **(A–D) Retrovirally transduced C3H10T1/2 cells (A, B) and primary MEFs (C, D) expressing Zfp521 or empty vector were cultured in osteogenic media containing β-glycerophosphate and ascorbic acid.** After 7 (10T1/2) or 18 (MEFs) d, cells were stained with alkaline phosphatase or Alizarin Red and osteogenic markers were measured by Q-PCR (Alpl, Alkaline phosphatase; Osx, osterix; Ocn, Osteocalcin; Col1A1, Type I collagen; Bsp: Bone sialoprotein). (E, F) MEFs from (C, D) differentiated with DMIR stained with oil red-O (E) and adipocyte markers were determined by Q-PCR (F) on day 8.
(TIF)

Figure S3 **(A) Adipocyte gene expression of BAT from** ***Zfp521^+/+^*** **and** ***Zfp521^−/−^*** **embryos were measured by Q-PCR. Data presented as mean ± SD,** ***n*** = 4, * ***p*** **<0.05.** (B, C) shZfp521 and shScr expressing F442A cells were mixed and injected into nude mice. Resulting fat pads were dissected and subjected to HE staining (B) and immunoblotting of C/EBP and FABP4 (*n* = 4) (C). (D) F442A cells expressing sh521 or shScr were mixed at the indicated ratios and subjected to genomic DNA isolation. 50 ng of genomic DNA was used as template for Q-PCR to detect variant and invariant hairpins. Relative fold ratio was determined by normalizing samples to the 1∶1 mixture of cells 10 wk after transplantation. (E, F) C3H10T1/2 cells were transduced with pMSCV, PPARγ, or C/EBPα in the presence or absence of Zfp521 as indicated prior to differentiation with DMIR and staining with oil red-O and adipocyte gene expression was measured by Q-PCR. (G) Immortalized *Pparg^+/−^* and *Pparg^−/−^* MEFs were transduced with retrovirus bearing shScr or shZfp521 and stained with oil red-O. (H, J) Zfp521 mRNA was measured by Q-PCR prior to differentiation. (I, K) Cells from (H and J) were differentiated with DMIR and adipocyte genes were measured by Q-PCR. Data presented as mean ± SD, *n* = 3, *p<0.05.
(TIF)

Figure S4 **(A) 3T3-L1 preadipocytes were transduced with retrovirus expressing sh521, shScr, Zfp521, or empty vector.** After puromycin selection, RNA was collected and submitted for analysis using Affymetrix arrays. The Venn diagram shows the number of genes down-regulated by sh521 (sh521/shScr<0.7-fold) and up-regulated by Zfp521 (Zfp521/pMSCV>1.5-fold). The heat map corresponds to genes in the intersecting set. (B) HeLa cells were transfected with HA-Zfp521, Myc-Ebf1, or empty vector as indicated. After 24 h, cells were harvested and immunoprecipitation was performed with anti-Myc beads. 10% input and the SDS eluate were subjected to Western blotting with antibodies against HA or Myc. WCL, whole cell lysate. (C) Immortalized *Zfp521^+/+^* and *Zfp521^−/−^* MEFs were transduced with a retrovirus expressing Flag-Ebf1; expression was measured by western blotting with anti-Flag prior to differentiation. (D) Schematic depicting the protein structure of WT Zfp521, Zfp521ΔZF27-30, and Zfp521Δ13aa. Individual zinc fingers are depicted as black bars. (E) 3T3-L1 preadipocytes were transfected with Myc-Ebf1 and either Flag-Zfp521WT or Flag-Zfp521Δ27-30. 24 h after transfection, cells were harvested and immunoprecipitation was performed using anti-Myc beads. 10% input and the SDS eluate were subjected to Western blotting with antibodies against Zfp521, Flag, or Myc. (F) 3T3-L1 preadipocytes were co-transfected with vectors expressing Zfp521, Zfp521Δ27-30, Zfp521Δ13aa, Myc-Ebf1, and Cebpa-promoter. 24 h after transfection, luciferase activity was determined. Data presented as mean ± SD, *n* = 6, *p<0.05. (G) C3H10T1/2 cells were transduced with a retrovirus expressing Zfp521 WT, Zfp521ΔZF27-30, Zfp521Δ13aa, or empty pMSCV vector. Protein expression of Zfp521 was determined by immunoblotting.
(TIF)

Figure S5 **(A–C) 293T cells were transiently transfected with 2HA-Zfp521 (521), 3Flag-Zfp423 (423), and 6Myc-Ebf1 (Ebf1).** After 48-h transfection, cell lysates were subjected to co-immunoprecipitation with α-Flag, α-HA, or α-Myc antibodies as indicated and 5% input were blotted with α-Flag, α-HA, or α-Myc antibodies.
(TIF)

Figure S6 **Gene expression was measured by Q-PCR before confluency (70%) and after adding DMIR in C3H10T1/2 cells at indicated time points.**
(TIF)

Table S1 **Primer sequences.**
(DOC)

## Abbreviations

ASC: adipose stromal cell
Ebf1: early B cell factor 1
GFP: green fluorescent protein
HE: hematoxylin–eosin
MEF: mouse embryonic fibroblast
SD: standard deviation
