# Supplementary figures and images for: Regulation of Early Adipose Commitment by Zfp521
Source: PLoS Biol. 2012 Nov 27;10(11):e1001433. doi: 10.1371/journal.pbio.1001433 (PMC3507953; doi:10.1371/journal.pbio.1001433)

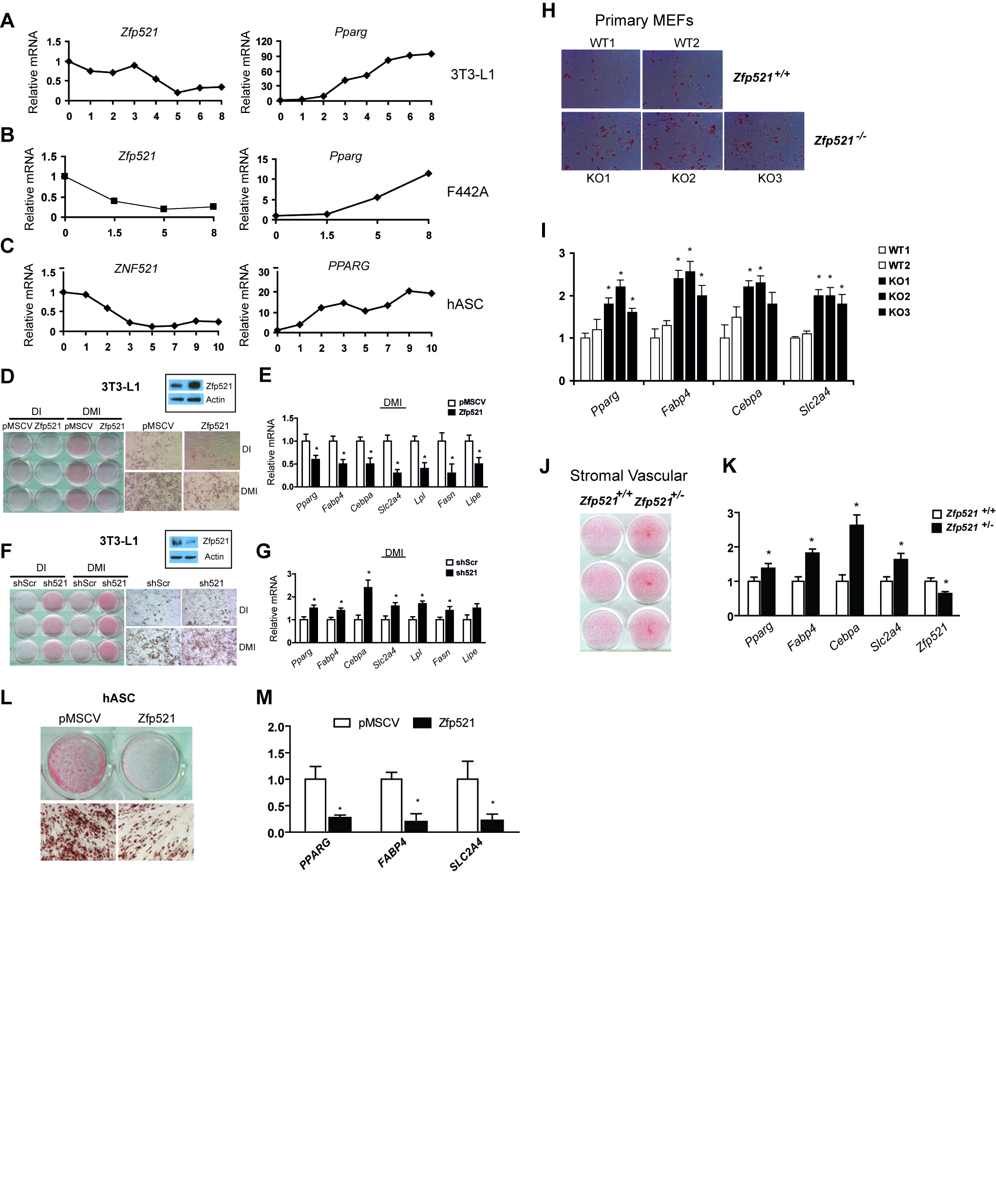

Supplement: Figure S1 — (A, B) 3T3-L1 cells (A), 3T3-F442A cells (B), and human ASCs (C) were differentiated and RNA isolated at the indicated time points. Gene expression ofZfp521/ZNF521 and Pparg/PPARG was measured by Q-PCR. Relative expression was normalized to cyclophilin. Data shown as mean of three biological replicates. (C, D) Retrovirally transduced 3T3-L1 cells expressing Zfp521, empty vector, shRNA specific forZfp521 (sh521) or a scrambled hairpin (Scr) were differentiated with DM or DMI and stained with oil red-O (D, F) and adipocyte markers were determined by Q-PCR (E, G) on day 8. (H, I) Primary MEFs were generated from Zfp521+/+ and Zfp521−/−embryos and differentiated with DMIR stained with oil red-O (H) and adipocyte markers were determined by Q-PCR (I) on day 8. (J, K) Stromal vascular cells were isolated from epididymal adipose tissue of Zfp521+/+ and Zfp521+/− mice. Cells were differentiated with DMIR, stained with oil red-O (J) and adipocyte gene expression was determined by Q-PCR (K) on day 8. Data presented as mean ± SD, n = 3, *p<0.05. (L, M) Human ASCs were transfected with Zfp521 or pMSCV and differentiated with DMIR. Adipogenesis was assessed with oil red-O and gene expression analysis on day 7. (TIF) [file pbio.1001433.s001.tif]

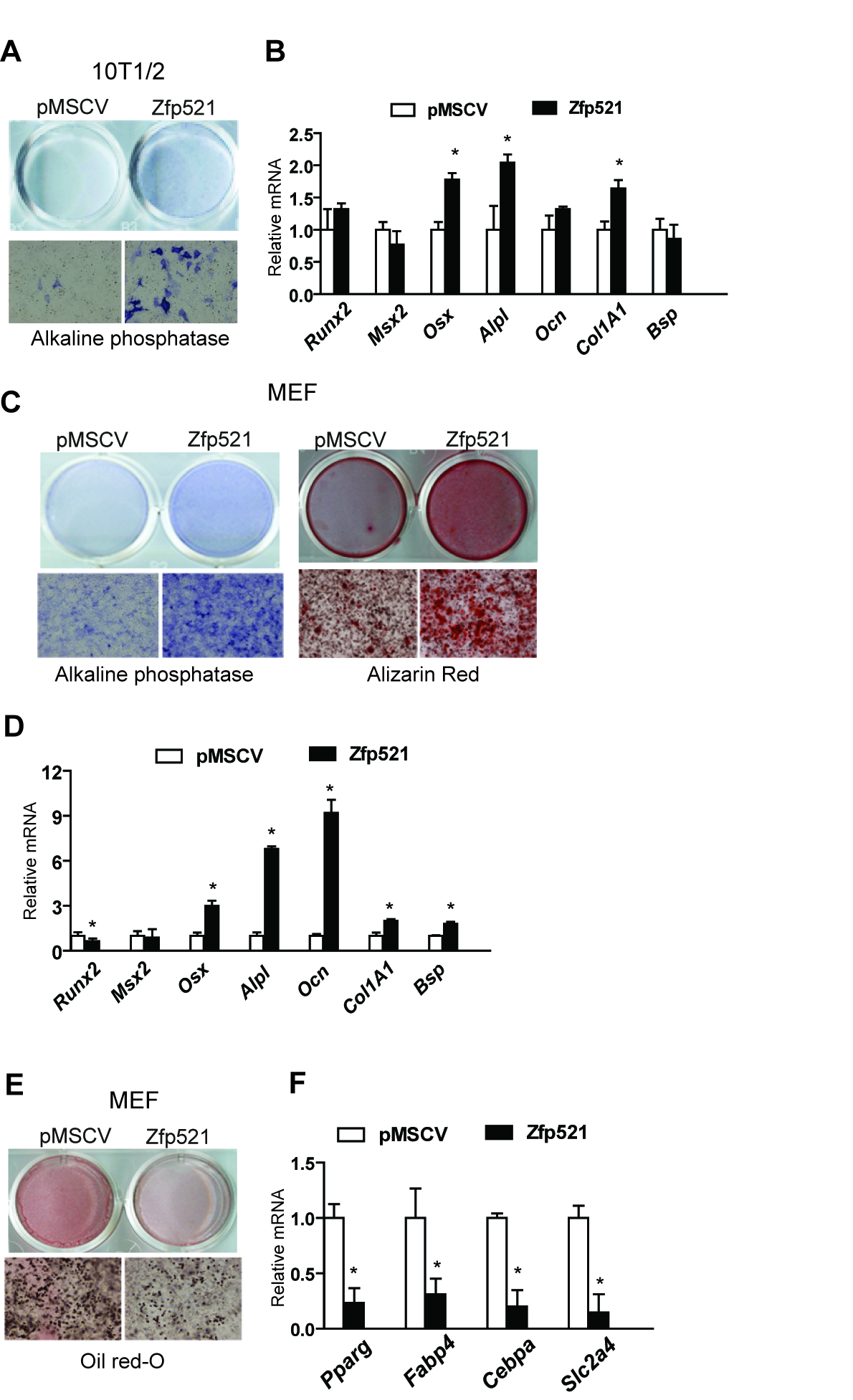

Supplement: Figure S2 — (A–D) Retrovirally transduced C3H10T1/2 cells (A, B) and primary MEFs (C, D) expressing Zfp521 or empty vector were cultured in osteogenic media containing β-glycerophosphate and ascorbic acid. After 7 (10T1/2) or 18 (MEFs) d, cells were stained with alkaline phosphatase or Alizarin Red and osteogenic markers were measured by Q-PCR (Alpl, Alkaline phosphatase; Osx, osterix; Ocn, Osteocalcin; Col1A1, Type I collagen; Bsp: Bone sialoprotein). (E, F) MEFs from (C, D) differentiated with DMIR stained with oil red-O (E) and adipocyte markers were determined by Q-PCR (F) on day 8. (TIF) [file pbio.1001433.s002.tif]

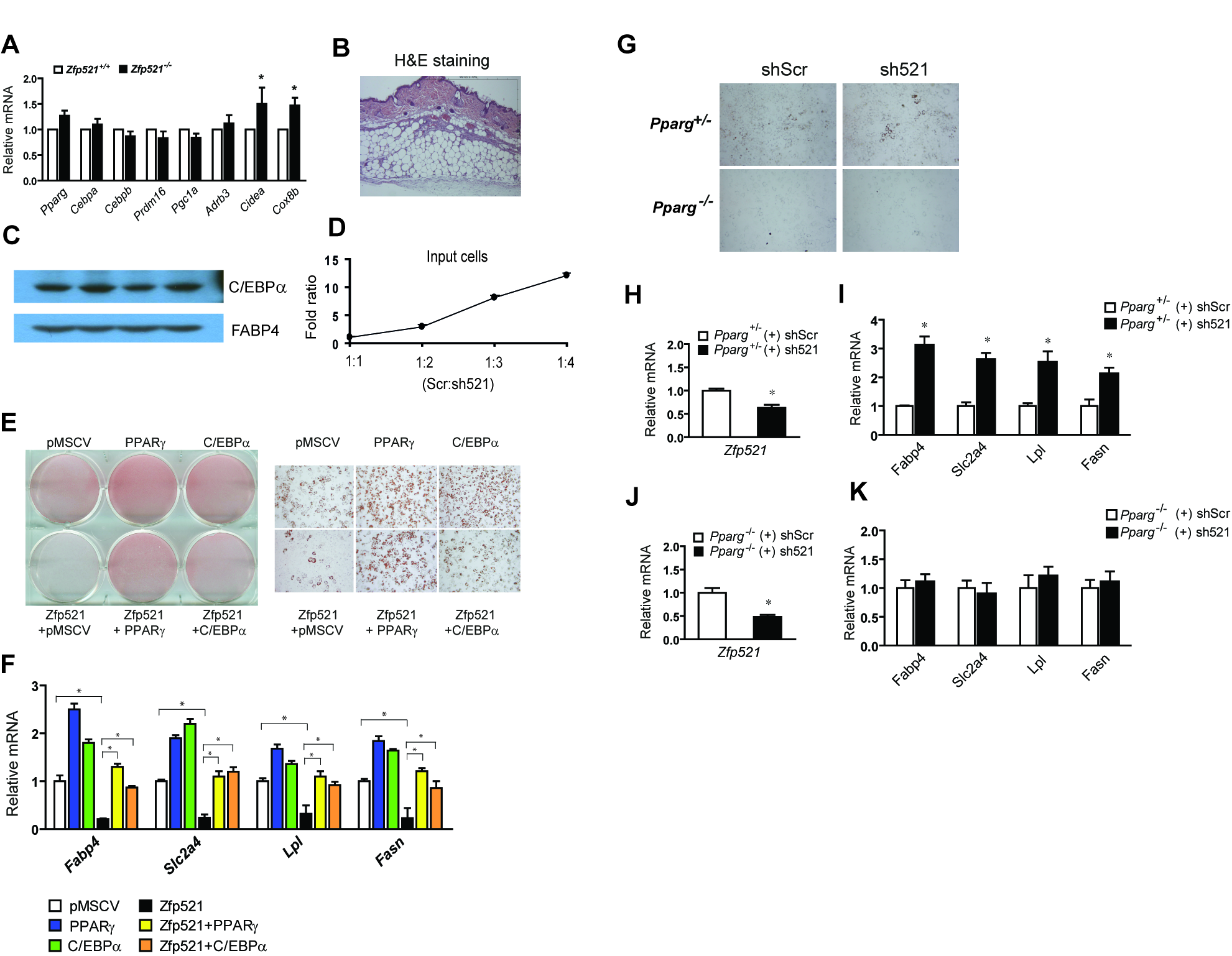

Supplement: Figure S3 — (A) Adipocyte gene expression of BAT from Zfp521+/+ and Zfp521−/− embryos were measured by Q-PCR. Data presented as mean ± SD, n = 4, * p <0.05. (B, C) shZfp521 and shScr expressing F442A cells were mixed and injected into nude mice. Resulting fat pads were dissected and subjected to HE staining (B) and immunoblotting of C/EBP and FABP4 (n = 4) (C). (D) F442A cells expressing sh521 or shScr were mixed at the indicated ratios and subjected to genomic DNA isolation. 50 ng of genomic DNA was used as template for Q-PCR to detect variant and invariant hairpins. Relative fold ratio was determined by normalizing samples to the 1∶1 mixture of cells 10 wk after transplantation. (E, F) C3H10T1/2 cells were transduced with pMSCV, PPARγ, or C/EBPα in the presence or absence of Zfp521 as indicated prior to differentiation with DMIR and staining with oil red-O and adipocyte gene expression was measured by Q-PCR. (G) Immortalized Pparg+/− and Pparg−/− MEFs were transduced with retrovirus bearing shScr or shZfp521 and stained with oil red-O. (H, J) Zfp521 mRNA was measured by Q-PCR prior to differentiation. (I, K) Cells from (H and J) were differentiated with DMIR and adipocyte genes were measured by Q-PCR. Data presented as mean ± SD, n = 3, *p<0.05. (TIF) [file pbio.1001433.s003.tif]

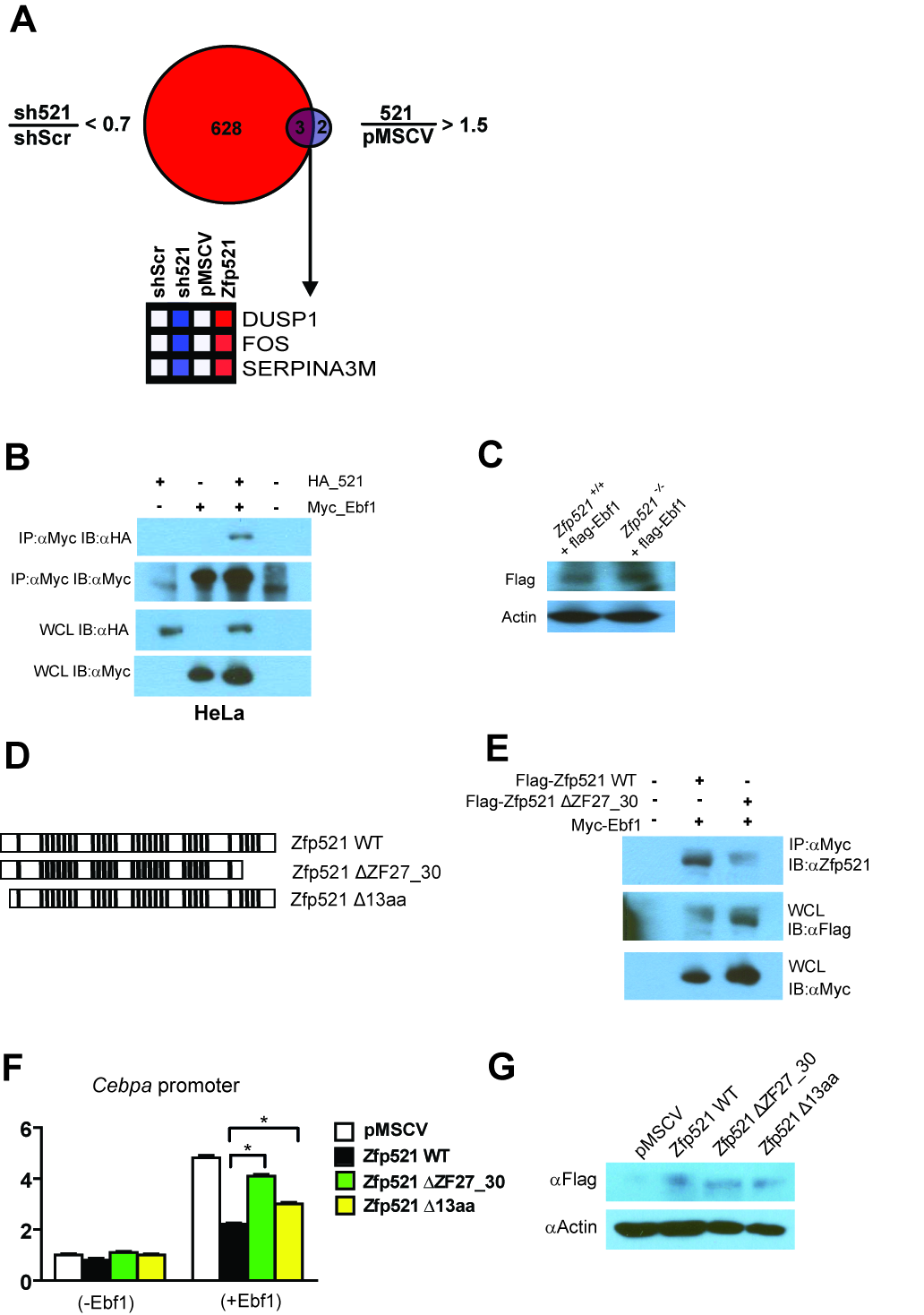

Supplement: Figure S4 — (A) 3T3-L1 preadipocytes were transduced with retrovirus expressing sh521, shScr, Zfp521, or empty vector. After puromycin selection, RNA was collected and submitted for analysis using Affymetrix arrays. The Venn diagram shows the number of genes down-regulated by sh521 (sh521/shScr<0.7-fold) and up-regulated by Zfp521 (Zfp521/pMSCV>1.5-fold). The heat map corresponds to genes in the intersecting set. (B) HeLa cells were transfected with HA-Zfp521, Myc-Ebf1, or empty vector as indicated. After 24 h, cells were harvested and immunoprecipitation was performed with anti-Myc beads. 10% input and the SDS eluate were subjected to Western blotting with antibodies against HA or Myc. WCL, whole cell lysate. (C) Immortalized Zfp521+/+ and Zfp521−/− MEFs were transduced with a retrovirus expressing Flag-Ebf1; expression was measured by western blotting with anti-Flag prior to differentiation. (D) Schematic depicting the protein structure of WT Zfp521, Zfp521ΔZF27-30, and Zfp521Δ13aa. Individual zinc fingers are depicted as black bars. (E) 3T3-L1 preadipocytes were transfected with Myc-Ebf1 and either Flag-Zfp521WT or Flag-Zfp521Δ27-30. 24 h after transfection, cells were harvested and immunoprecipitation was performed using anti-Myc beads. 10% input and the SDS eluate were subjected to Western blotting with antibodies against Zfp521, Flag, or Myc. (F) 3T3-L1 preadipocytes were co-transfected with vectors expressing Zfp521, Zfp521Δ27-30, Zfp521Δ13aa, Myc-Ebf1, and Cebpa-promoter. 24 h after transfection, luciferase activity was determined. Data presented as mean ± SD, n = 6, *p<0.05. (G) C3H10T1/2 cells were transduced with a retrovirus expressing Zfp521 WT, Zfp521ΔZF27-30, Zfp521Δ13aa, or empty pMSCV vector. Protein expression of Zfp521 was determined by immunoblotting. (TIF) [file pbio.1001433.s004.tif]

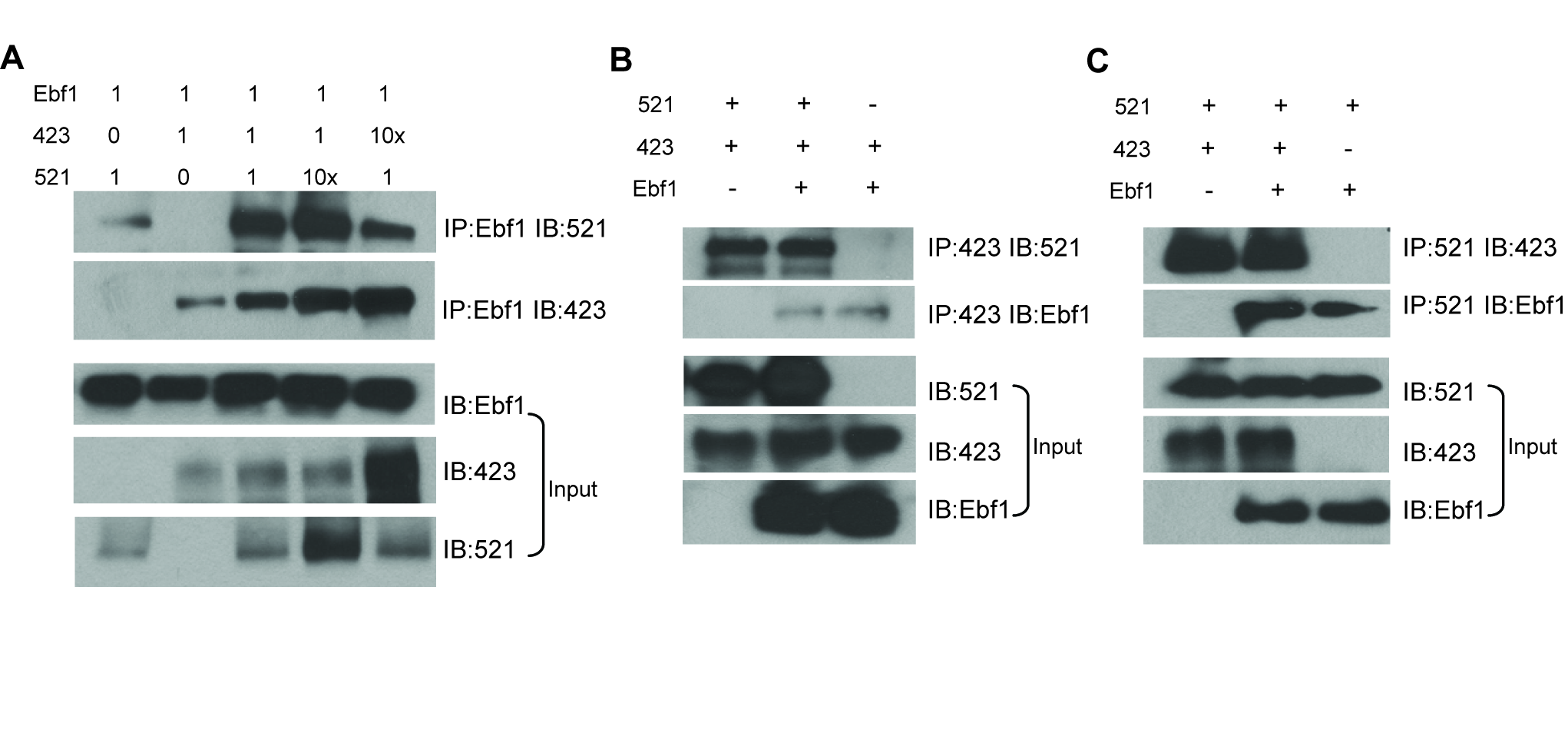

Supplement: Figure S5 — (A–C) 293T cells were transiently transfected with 2HA-Zfp521 (521), 3Flag-Zfp423 (423), and 6Myc-Ebf1 (Ebf1). After 48-h transfection, cell lysates were subjected to co-immunoprecipitation with α-Flag, α-HA, or α-Myc antibodies as indicated and 5% input were blotted with α-Flag, α-HA, or α-Myc antibodies. (TIF) [file pbio.1001433.s005.tif]

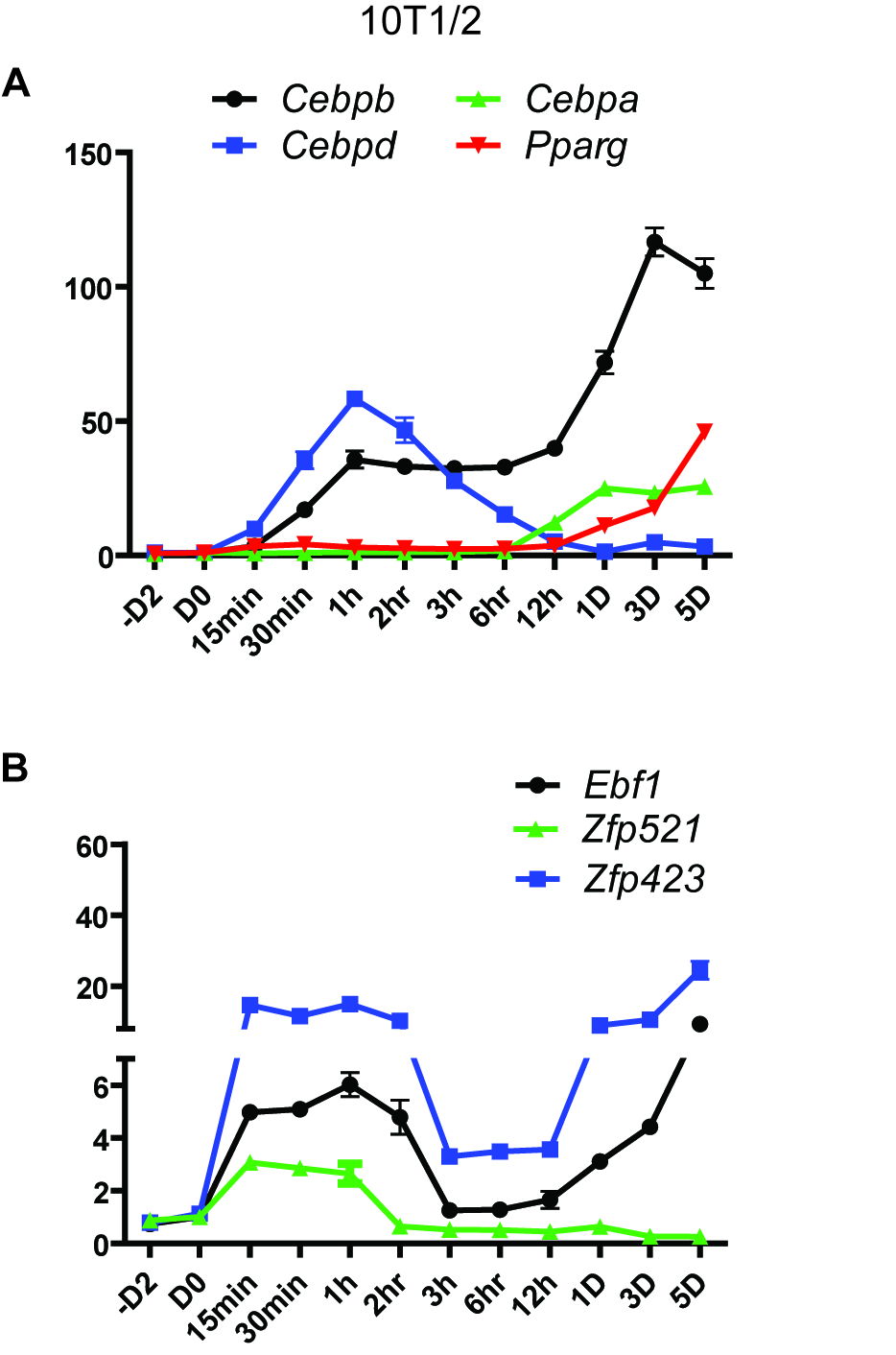

Supplement: Figure S6 — Gene expression was measured by Q-PCR before confluency (70%) and after adding DMIR in C3H10T1/2 cells at indicated time points. (TIF) [file pbio.1001433.s006.tif]
