# Supplementary material for: Regulation of Early Adipose Commitment by Zfp521
Source: PLoS Biol. 2012 Nov 27;10(11):e1001433. doi: 10.1371/journal.pbio.1001433 (PMC3507953; doi:10.1371/journal.pbio.1001433)
Supplement: Table S1 — Primer sequences. (DOC) [file pbio.1001433.s007.doc]

**Supplemental Table S1. Primer sequences**

| **Primers** | **Sequences (5’>3’)** |
| --- | --- |
| Vector invariant-F | CTTGGGTAGTTTGCAGTTT |
| Vector invariant-R | GCATGCTCCAGACTGCCTT |
| shZfp521/Scr variant-F | CTTGGGTAGTTTGCAGTTT |
| shZfp521 variant-R | AAGAAGACGAAGGTGTGCA |
| Scr variant-R (m) | GAGGGACTAAAGATAGGAC |
| Cyclophilin-F (m) | TTCCTCCTTTCACAGAATTATTCCA |
| Cyclophilin-R (m) | CCGCCAGTGCCATTATGG |
| Cebpa-F (m) | GACCATTAGCCTTGTGTGTACTGTATG |
| Cebpa-R (m) | TGGATCGATTGTGCTTCAAGTT |
| Pparg-F (m) | CAAGAATACCAAAGTGCGATCAA |
| Pparg-R (m) | GAGCTGGGTCTTTTCAGAATAATAAG |
| Slc2a4-F (m) | TCATTGTCGGCATGGGTTT |
| Slc2a4-R (m) | CGGCAAATAGAAGGAAGACGTA |
| Pref1-F (m) | TTCGGGCTTGCACCTCAA |
| Pref1-R (m) | GGAGCATTCGTACTGGCCTTT |
| Lpl-F (m) | ACAAAGTGTTCCATTACCAAGTCAAG |
| Lpl-R (m) | GTGCCGTACAGAGAAATTTCGA |
| Fasn-F (m) | GTTTTGAGGGATGCCATGCT |
| Fasn-R (m) | GGGTTGCCCTGTCAAGGTT |
| Lipe-F (m) | CCGCTGACTTCCTGCAAGAG |
| Lipe-R (m) | CTGGGTCTATGGCGAATCGG |
| Fabp4-F (m) | CTTCAAACTGGGCGTGGAA |
| Fabp4-R (m) | CTAGGGTTATGATGCTCTTCACCTT |
| Ebf1-F (m) | TGCTGGTCTGGAGTGAGTTGA |
| Ebf1-R (m) | CCACCACACCAGGGATGTG |
| Zfp521-F (m,h) | GGCTGTTCAAACACAAGCG |
| Zfp521-R (m,h) | GCACATTTATATGGCTTGTTG |
| Zfp423-F (m,h) | GATCACTGTCAGCAGGACTT |
| Zfp423-R (m,h) | TGCCTCTTCAAGTAGCTCA |
| Cyclophilin-F (h) | ACGGGTCCTGGCATCTTG |
| Cyclophilin-R (h) | TGCCATCCAACCACTCAGTCT |
| Pparg-F (h) | TGACAGCGACTTGGCAATATTTATT |
| Pparg-R (h) | TTGTAGCAGGTTGTCTTGAATGTCT |
| Ebf1-F (h) | **TGTGCCGAGTCTTGCTCACA** |
| Ebf1-R (h) | **TCCGCATGTCACGTGGGTTT** |
| Cebpa-ChIP-F | **TCTCTCTCCACTA GCACTATGC** |
| Cebpa-ChIP-R | **AACTGGCTCGCGCCCGCGCA** |
| Control-ChIP-F | **GTTTTTAGCCGTCCTCTTG** |
| Control-ChIP-R | **GTCTCGTTCACCCTCCACCT** |
| Peak1-ChIP-F | **AATGATGAAATTCAGTTGCTATGC** |
| Peak1-ChIP-R | **AAGATTTTCAAGATTGCTGGTAGG** |
| Peak2-ChIP-F | **TTGCGTGTCCCCAAGCGGTC** |
| Peak2-ChIP-R | **GCAATGCATTAAGCAATCTCCCTGC** |
| Peak3-ChIP-F | **ACGCTTGCCAAAAGGGAAGAAAGG** |
| Peak3-ChIP-R | **TCTCCACATCTGGTGCTTTGCTC** |

*(m): mouse, (h): human
